# Upregulation of Heme Oxygenase-1 Endues Immature Dendritic Cells With More Potent and Durable Immunoregulatory Properties and Promotes Engraftment in a Stringent Mouse Cardiac Allotransplant Model

**DOI:** 10.3389/fimmu.2018.01515

**Published:** 2018-07-02

**Authors:** Yue Zhao, Yu Jia, Lu Wang, Song Chen, Xia Huang, Bingyang Xu, Guangyuan Zhao, Ying Xiang, Jun Yang, Gang Chen

**Affiliations:** ^1^Institute of Organ Transplantation, Tongji Hospital, Huazhong University of Science and Technology, Wuhan, China; ^2^Sichuan Cancer Hospital and Institute, Sichuan Cancer Center, Chengdu, China; ^3^School of Medicine, University of Electronic Science and Technology of China, Chengdu, China; ^4^Department of Nephrology, Tongji Hospital, Tongji Medical College of Huazhong University of Science and Technology, Wuhan, China; ^5^Key Laboratory of Organ Transplantation, Ministry of Education, Wuhan, China; ^6^Key Laboratory of Organ Transplantation, Ministry of Public Health, Wuhan, China

**Keywords:** dendritic cells, heme oxygenase-1, immunoregulation, heart transplantation, mouse

## Abstract

Heme oxygenase-1 (HO-1) is critical for the ability of immature dendritic cells (imDCs) to suppress T-cell responses. Induction of high HO-1 expression may markedly improve the tolerogenic capacity of imDCs. Here, we generated bone marrow-derived DCs (BMDCs) from BALB/c mice with low doses of GM-CSF and IL-4. The adherent BMDCs were obtained as imDCs. Upregulation of HO-1 in imDCs (HO-1^hi^-imDCs) was achieved by cobalt protoporphyrin treatment. HO-1^hi^-imDCs proved to be more maturation-resistant than conventional imDCs, with an enhanced ability to inhibit allogeneic T-cell proliferation stimulated by anti-CD3/CD28 antibodies. When donor-derived DC adoptive transfer was performed in a stringent mouse cardiac allotransplant model, the extent of graft prolongation observed with HO-1^hi^ imDCs was superior to that obtained with conventional imDCs. T-cell activation and proliferation in cardiac allograft recipients was more strongly suppressed in the HO-1^hi^ imDC transfusion group than that in the untreated imDC group. Furthermore, donor HO-1^hi^ imDCs were able to maintain a status of high HO-1 expression and survived longer in the recipient spleens than did untreated imDCs after adoptive transfer. *In vitro*-generated HO-1^hi^ imDCs had an enhanced tolerogenic capacity to modulate alloimmune responses both *in vitro* and *in vivo*, and thus may offer a novel antigen-specific and cost-effective strategy to induce transplant tolerance.

## Introduction

As the professional antigen-presenting cells, dendritic cells (DCs) do not always induce pro-inflammatory immune responses. The potential of DCs to either stimulate or inhibit immune responses is directly related to their maturation status (1). Inflammatory T-cell responses are mediated by mature dendritic cells (mDCs) that express high levels of cell-surface major histocompatibility complex (MHC) class II proteins and costimulatory molecules, whereas T-cell tolerance can be induced by immature dendritic cells (imDCs) that express much lower levels of these antigen-presenting and costimulatory molecules (2, 3). This tolerance mainly results from the deletion of T cells, the induction of regulatory T cells (Tregs) and anergic T cells, the expression of immunomodulatory molecules (e.g., PD-L1, PD-L2, hemeoxygenase-1, HLA-G, CD95L, galectin-1, and DC-SIGN), and the production of immunosuppressive factors (e.g., IL-10, TGF-β, IDO, IL-27, NO, and CTLA4) (4–7). Adoptive transfer of *in vitro*-generated donor or recipient (autologous) imDCs has been evaluated in rodent allotransplant models (8–11). In these studies, both sources have been shown to be able to prevent or delay allograft rejection. The apparent pro-tolerogenic properties of imDCs make them attractive candidates for use in promoting immune tolerance after clinical solid-organ transplantation.

A major concern with the use of imDCs is the unpredictable maturation process that follows their adoptive infusion into an *in vivo* system, particularly into an inflammatory environment such as that occurring in the situation of organ transplantation (6, 12). In addition, donor-derived imDCs are usually short-lived after they are intravenously injected into a recipient, potentially also limiting their *in vivo* immunomodulatory effects (6, 9, 13). In an effort to generate better tolerogenic imDCs (tol-DCs) with maturation resistance, *in vitro*-propagated DCs have been manipulated through either genetic engineering technology to express immunosuppressive molecules (such as IL-10, TGF-β, IDO, and CTLA4) or pharmacological modifications (including rapamycin, mitomycin-C, and dexamethasone) to enhance DC tolerogenicity (7, 14). These modified tol-DCs all have improved graft survival (14).

Heme oxygenase-1 (HO-1) is an inducible heme-catabolizing enzyme that has been demonstrated to be an anti-apoptosis, anti-inflammatory, immunosuppressive molecule (15, 16). In a rat and nonhuman primate study, HO-1 has been reported to play a critical role in the active suppression of T-cell responses by tol-DCs, and tol-DCs lose their immunoregulatory effects when HO-1 is blocked (17). Thus, induction of HO-1 high expression may markedly improve the tolerogenic capacity of tolDCs. Another *in vitro* study on human and rat imDCs has demonstrated that induction of high HO-1 expression can inhibit lipopolysaccharide (LPS)-induced DC maturation and decrease the capacity of rat and human DCs to stimulate allogeneic T-cell proliferation (18). However, it remains to be determined whether *in vitro*-generated imDCs with induced high expression of HO-1 (HO-1^hi^-imDCs) can be more potent in actively inhibiting allogeneic T-cell activation and proliferation or have an enhanced *in vivo* immunoregulatory capacity to extend allograft survival after adoptive transfer.

In the present study, we have obtained HO-1^hi^-imDCs by *in vitro* treatment of mouse adherent bone marrow-derived DCs (BMDCs) with cobalt protoporphyrin (CoPP) and have demonstrated, for the first time, that when compared to conventional imDCs, HO-1^hi^-imDCs are more potent and maturation-resistant immunoregulatory DCs that can survive longer, with sustained HO-1 high-expression status in allogeneic recipients after adoptive transfer. These improvements together result in a significant prolongation of allograft survival in a stringent mouse cardiac allotransplant model.

## Materials and Methods

### Animals

Male BALB/c (H2^d^) and C57BL/6 (H2^b^) mice (6–8 weeks of age) were purchased from HFK Biosciences (Beijing, China). Animals were maintained according to the *Guide for the Care and Use of Laboratory Animals* of the National Institutes of Health, and the protocol was approved by the Ethical Committee on Animal Experiments of Tongji Medical College, Huazhong University of Science and Technology.

### Monoclonal and Polyclonal Antibodies

APC-conjugated anti-mouse CD11c; PE-conjugated anti-mouse MHC-II and CD11b; PE-Cy5-conjugated anti-mouse CD86; FITC-conjugated anti-mouse CD40, CD80, CD83, and GR-1, and PE-conjugated anti-mouse IFN-γ monoclonal antibodies were purchased from eBioscience (San Diego, CA, USA). Polyclonal anti-HO-1 Ab was purchased from Enzo Life Sciences (Lausen, Switzerland). Polyclonal anti-tubulin Ab was purchased from Beyotime (Shanghai, China).

### Reagents

Mouse rGM-CSF and rIL-4 were purchased from Peprotech (Rocky hill, NJ, USA). CFSE, 2-ME, l-glutamine, and sodium pyruvate were purchased from Invitrogen (Carlsbad, CA, USA). CoPP and tin protoporphyrin (SnPP) were purchased from Phophyrin Products (Logan, UT, USA). LPS was purchased from Sigma-Aldrich (St. Louis, MO, USA). Mouse IL-10 and IFN-γ ELISA kits were purchased from eBioscience (San Diego, CA, USA).

### Cell Preparations

#### Bone Marrow-Derived DCs

Murine DCs were generated from BM according to published methods, with modifications (19). In brief, BM cells were isolated from femurs and tibia of BALB/c mice. BM cells were differentiated *in vitro* toward imDCs for 9 days in the presence of 20 ng/ml murine rGM-CSF and 10 ng/ml murine rIL-4 in cRPMI 1640 (RPMI supplemented with 10% heat-inactivated FBS, 100 U/ml penicillin, 100 µg/ml streptomycin, 50 µM 2-ME, 2 mM l-glutamine, and 1 mM sodium pyruvate). The medium was refreshed on days 4 and 7. On day 9, adherent immature BMDCs were collected and used. Non-adherent BMDCs treated with 1 µg/ml LPS for 24 h were collected as mDCs.

Metalloprotoporphyrins were dissolved in 0.2 N NaOH, neutralized with 0.2 N HCL, adjusted to 0.5 mg/ml, and sterilized by filtration. Murine BMDCs were pulsed for 2 h with 50 µM CoPP or SnPP, an inducer or an inhibitor of HO-1, respectively. The cells were then washed twice and cultured for 16 h before use.

#### T Cells

Purified lymphocytes were prepared from buffy-coat preparations of homogenized lymph nodes or spleens from C57BL/6 or BALB/c mice by density gradient centrifugation with murine lymphocyte isolation medium (TBD, China). In mixed leukocyte reaction (MLR) experiments, purified CD3^+^ T cells were prepared by positive selection using a CD3^+^ T-cell isolation kit. The purity of the CD3^+^ populations was determined as >95% by flow cytometry. To detect lymphocyte proliferation, CD3^+^ T cells were labeled with 1 µM CFSE.

### Phenotyping by FACS

The obtained murine DCs were stained directly with anti-mouse conjugated Abs against the following: CD11c, CD11b, MHC-II, CD80, CD86, CD40, CD83, and GR-1; they were compared to matching isotype controls. Cells were analyzed using a FACSCalibur with Cellquest Pro 6.0 software (BD Biosciences, USA).

### Measurements of HO-1 Expression

#### Western Blotting

Murine DCs extracts were boiled, electrophoresed on a sodium dodecyl sulfate polyacrylamide gel for 30 min at 80 V, followed by 60 min at 110 V. Membranes were blocked in PBS containing 5% BSA and, after washing, incubated overnight with rabbit anti-HO-1 polyclonal Ab in PBS containing 0.5% Tween-20. After washing, the membranes were incubated for 2.5 h with HRP-conjugated goat anti-rabbit IgG (Zhongshan Golden Bridge Biotechnology Co., Ltd., China; 1:8,000). Tubulin was used as an intrinsic quality control. The specific bands were visualized using the ECL reagent (Beyotime, Beijing, China) and recorded by a Molecular Imager ChemiDoc XRS System (Bio-Rad, USA).

#### FACS

Murine DCs were fixed in acetone, permeabilized, and stained with rabbit anti-HO-1 primary polyclonal antibody, followed by a FITC-conjugated goat anti-rabbit secondary mAb. Cells were analyzed using a FACSCalibur with Cellquest Pro 6.0 software (BD Biosciences, USA).

#### Immunofluorescent Staining

Dendritic cells were washed twice with PBS and fixed with 4% polyoxymethylene for 30 min, then permeabilized with 0.1% Triton X-100 in PBS for 30 min at room temperature and blocked for 1 h with 2% BSA at 37°C. To detect HO-1, cells were incubated overnight with rabbit anti-HO-1 primary polyclonal Ab and APC-conjugated anti-mouse CD11c mAb at 4°C. The cells were then washed with PBS, and a secondary antibody (FITC-conjugated goat anti-rabbit secondary mAb) was applied for 30 min at 37°C. The nuclei were stained with DAPI (Beyotime, Shanghai, China; 1:1,000). Color images were captured under a confocal imaging system (Perkin Elmer, Waltham, MA, USA).

### Lymphocyte Inhibition Assays

#### FACS

Plate-bound murine anti-CD3ε (2 µg/ml) and soluble anti-CD28 Abs (2 µg/ml) were used to stimulate 1 µM CFSE-labeled lymphocytes (2 × 10^5^) from C57BL/6 mice in 96-well *U*-bottom plates. Graded doses of imDCs with different pretreatment or LPS-stimulated mDCs from BALB/c were added to the co-culture medium (DC-to-lymphocyte ratios of 1:4, 1:8, 1:16, and 1:32). After 3 days, lymphocytes were harvested and labeled with APC-conjugated anti-mouse CD4 mAb and APC-conjugated anti-mouse CD8 mAb, respectively. The proliferation of CD4^+^ and CD8^+^ T cells was analyzed using a FACSCalibur with Cellquest Pro 6.0 software (BD Biosciences, USA).

#### ELISA

The amounts of IL-10 and IFN-γ in the cell culture supernatants harvested from the above T-cell proliferation system (DC-to-lymphocyte ratio of 1:16) were assessed using ELISA kits according to the manufacturer’s instructions.

### Heart Transplantation

BALB/c mice served as heart donors, and C57BL/6 mice as recipients. Heterotopic cardiac allografts were performed as described previously (20). In brief, the donor aorta and pulmonary artery were anastomosed to the recipient abdominal aorta and inferior vena cava, respectively. Recipients were untreated or intravenously injected with 5 × 10^6^ untreated, CoPP-pretreated, or SnPP-pretreated donor-derived imDCs 7 days before transplantation. Graft function was monitored by abdominal palpation daily until rejection, which was defined as total cessation and was confirmed by direct visualization of the allograft.

### Graft Histology

Cardiac allografts were harvested at day 7 posttransplant. Tissue samples were fixed in formalin and then embedded in paraffin. Four-micrometer sections were stained with hematoxylin and eosin (H&E) by standard methods. Microscopic tissue sections were examined by a pathologist, in a blinded fashion, for severity of rejection. Criteria for graft rejection included the presence of vasculitis, thrombosis, hemorrhage, and inflammatory cell infiltration.

### Cell Tracking After Adoptive Transfer

To analyze DC localization after an intravenous injection, BALB/c mice-derived imDCs labeled with CellVue Claret were injected into C57BL/6 mice *via* the tail vein. At 1, 7, or 14 days after adoptive transfer, the mesenteric lymph nodes and spleens were harvested. After being stained with DAPI (Beyotime, Shanghai, China; 1:1,000), tissue sections were observed under a confocal imaging system (Perkin Elmer, Waltham, MA, USA).

To investigate the survival of donor-derived DCs in recipient spleen and their expression of HO-1, untreated, CoPP-treated, or SnPP-treated BALB/c imDCs (5 × 10^6^) labeled with Cellvue Claret were adoptively transferred into C57BL/6 mice *via* the caudal vein. 7 or 14 days later, the spleens were harvested and then processed as frozen sections. The sections were incubated overnight with rabbit anti-HO-1 primary polyclonal Ab at 4°C and followed by a secondary antibody (FITC-conjugated goat anti-rabbit secondary mAb) for 30 min at 37°C. The nuclei were stained with DAPI (Beyotime, Shanghai, China; 1:1,000). The expression of HO-1 in Cellvue Claret-labeled DCs was evaluated by a confocal imaging system (Perkin Elmer, Waltham, MA, USA), viewing a minimum of five random microscopic fields.

### Alloreactive T-Cell Responses

#### IFN-γ Expression

To detect T-cell activation, isolated recipient splenocytes on day 7 posttransplant were stimulated for 4 h in the presence of 50 ng/ml PMA, 1 mg/ml ionomycin, and 2 mM monensin (all from Sigma-Aldrich) at 37°C under 5% CO_2_, and then, the cells were stained with PE-conjugated anti-mouse IFN-γ and APC-conjugated anti-mouse CD4 or CD8 mAb according to the manufacturer’s instructions. The cells were analyzed using a FACSCalibur with Cellquest Pro 6.0 software (BD Biosciences, USA). All flow cytometric analyses were performed using isotype-matched, irrelevant Ig as the negative control.

#### MLR Assay

Purified CD3^+^ T cells (2 × 10^5^ cells/well) from recipient spleens on day 7 posttransplant were labeled with CFSE and then cocultured with γ-irradiated (30 Gy) donor peripheral blood lymphocytes (2 × 10^5^ cells/well) using round bottom 96-well plates. Seven days later, lymphocytes were harvested, and their proliferation was analyzed using a FACSCalibur with Cellquest Pro 6.0 software (BD Biosciences, USA).

### Detection of Alloantibodies

The circulating anti-donor IgG and IgM in the recipients’ sera on day 7 posttransplant were evaluated by flow cytometry. In brief, BALB/c mouse splenocytes were isolated and incubated at 4°C for 30 min with sera (1:40 dilutions) from all experimental groups. The cells were washed and then incubated with APC-conjugated anti-mouse CD3, FITC-conjugated anti-mouse IgG, and PE-conjugated anti-mouse IgM (BD Biosciences, USA). The stained cells were analyzed by flow cytometry (FACSCalibur, BD Biosciences). Data are expressed as geometric mean fluorescence intensity (GMean), which represents the intensity of antibody binding. Naive sera from C57BL/6 mice were used as negative controls.

### Statistical Methods

Data are given as means ± SD. All of the statistical analyses were performed using GraphPad Prism V5.0 (GraphPad Software, USA). The significance of differences between the selected groups was evaluated using Student’s *t*-test. The Kaplan–Meier method was used to calculate survival curves, followed by a log-rank test. *P* values < 0.05 were considered statistically significant.

## Results

### Phenotype of Mouse BM-Derived DCs

Bone marrow-derived DCs were generated from mouse BM cell cultures with murine rGM-CSF plus rIL-4. After 9 days in culture, two major cell populations were obtained: adherent and non-adherent cells. Examining their phenotypes by FACS analysis (Figure S1 in Supplementary Material) revealed that the majority of both cell populations expressed CD11c (>90%) and CD11b (>75%). Fewer than 10% of the cells expressed CD83 and GR-1. Both cell populations expressed relatively low levels of CD40, CD80, CD86, and MHC-II molecules; however, the expression of these molecules was lower in the adherent population than in the non-adherent, indicating that the adherent BMDCs were less mature than the non-adherent BMDCs. Therefore, the adherent BMDCs were collected and used as imDCs for subsequent studies. Since the maturation of non-adherent BMDCs was more easily stimulated by LPS, non-adherent BMDCs treated with 1 µg/ml of LPS for 24 h were collected and used as mDCs in this study.

### CoPP Treatment Induces High Expression of HO-1 in BM-Derived imDCs

**Figure 1:**
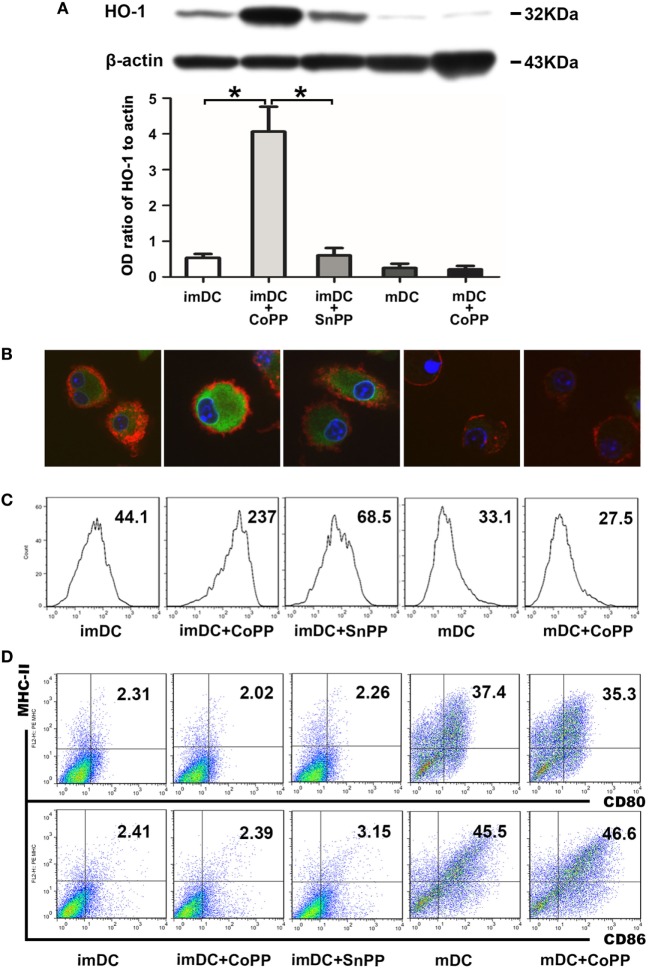
Cobalt protoporphyrin (CoPP) treatment induces HO-1 high expression in BM-derived immature dendritic cells (imDCs). Untreated, CoPP-pretreated, or SnPP-pretreated imDCs and untreated or CoPP-pretreated mDCs were used in the experiments. **(A)** HO-1 expression was measured by Western blotting. Anti-β-actin was used as a loading control. The corresponding densitometric analyses are shown as bar graphs. The mean ± SD is reported (*P < 0.05, *n* = 3 per group). **(B)** Immunofluorescent staining of HO-1 was performed on each group of dendritic cells (DCs). Confocal images shown are representative of three experiments with similar results (original magnification, 600×; red, CD11c; green, HO-1; blue, nuclei). **(C)** Flow cytometry analysis of HO-1 was performed on each group of DCs. Gmean values are shown. Similar results were obtained in three independent experiments. **(D)** The phenotype (MHC-II, CD80, and CD86) of each group of DCs was analyzed by flow cytometry. The results shown here are representative of three independent experiments.

To obtain DCs expressing high levels of HO-1, we treated *in vitro*-generated murine BM-derived imDCs or LPS-stimulated mDCs with 50 µM of CoPP for 2 h. HO-1 expression was evaluated by western blot analysis. Untreated imDCs had positive but not strong expression of HO-1, whereas mDCs only exhibited very weak expression of HO-1. The expression of HO-1 was significantly upregulated (by 5–10 times) after treatment of the cells with CoPP (*P* < 0.05 vs. the untreated imDC group or SnPP-treated imDC group). In contrast, CoPP treatment almost had no effect on the levels of HO-1 expression in mDCs (Figure 1A). These results were further confirmed by both immunofluorescent staining and FACS analysis for the intracellular expression of HO-1 (Figures 1B,C).

To investigate whether CoPP or SnPP treatment affects the DC phenotype, we evaluated the expression of several critical costimulatory molecules (MHC-II, CD80, and CD86) on the surface of imDCs or mDCs by FACS analysis: imDCs expressed low levels of MHC-II, CD80, and CD86, whereas mDCs expressed much higher levels of these costimulatory molecules (Figure 1D). After the treatment with CoPP or SnPP, we saw almost no change in the expression levels of MHC-II, CD80, and CD86 on either imDCs or mDCs.

### Upregulation of HO-1 Renders imDCs Refractory to LPS-Induced Maturation

**Figure 2:**
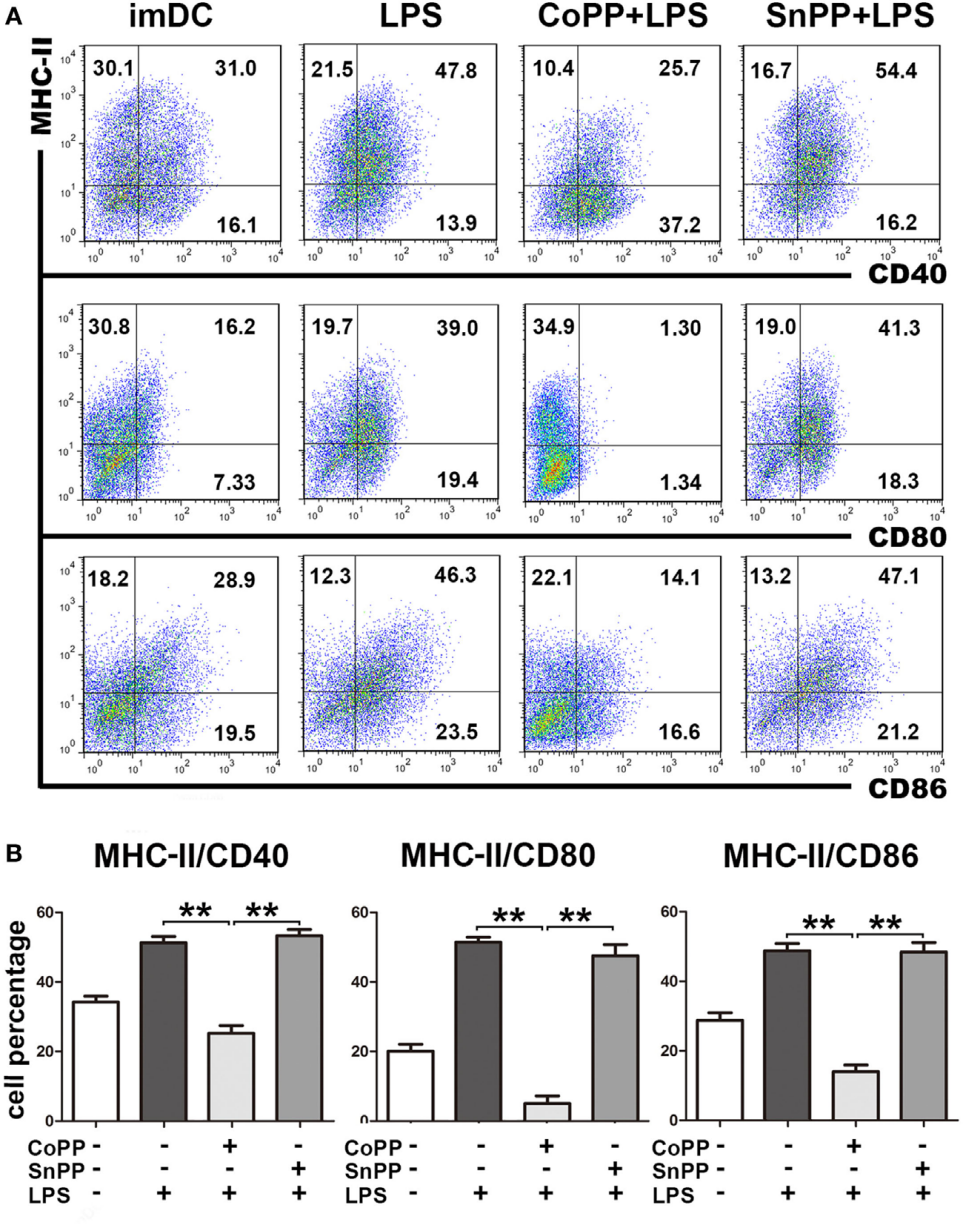
Upregulation of heme oxygenase-1 renders immature dendritic cells (imDCs) refractory to lipopolysaccharide (LPS)-induced maturation. Untreated, cobalt protoporphyrin-pretreated, or SnPP-pretreated imDCs were incubated with LPS to induce maturation for 24 h. Untreated imDCs without LPS stimulation were used as controls. The expression of MHC-II, CD40, CD80, and CD86 was assessed by FACS analysis. **(A)** Representative FACS results of three independent experiments. Numbers in quadrants indicate the percentage of positive cells. **(B)** Quantitative assessment of the percentages of MHC-II/CD40, MHC-II/CD80, and MHC-II/CD86 double-positive cells. The data shown are means ± SD (*n* = 3 per group; **P < 0.01).

To study the role of high HO-1 expression in the regulation of DC responses to pathogens, we pretreated imDCs with either CoPP or SnPP for 2 h, then incubated the cells with LPS to induce maturation for 24 h and assessed the expression of MHC-II, CD40, CD80, and CD86 by FACS analysis. We found that LPS was able to induce notable DC maturation of both untreated and SnPP-pretreated imDCs, as evidenced by a markedly increased expression of MHC-II, CD40, CD80, and CD86 (Figure 2). In contrast, significantly lower percentages of MHC-II/CD40, MHC-II/CD80, and MHC-II/CD86 double-positive cells were observed in CoPP-pretreated imDCs than in untreated or SnPP-pretreated imDCs after incubation with LPS (*P* < 0.01) (Figure 2), indicating that the induction of high HO-1 expression in imDCs resulted in a blockade of DC phenotypic maturation induced by LPS.

### Upregulation of HO-1 Enhances the Ability of imDCs to Inhibit Allogeneic T-Cell Proliferation Stimulated by Anti-CD3/CD28 Abs

**Figure 3:**
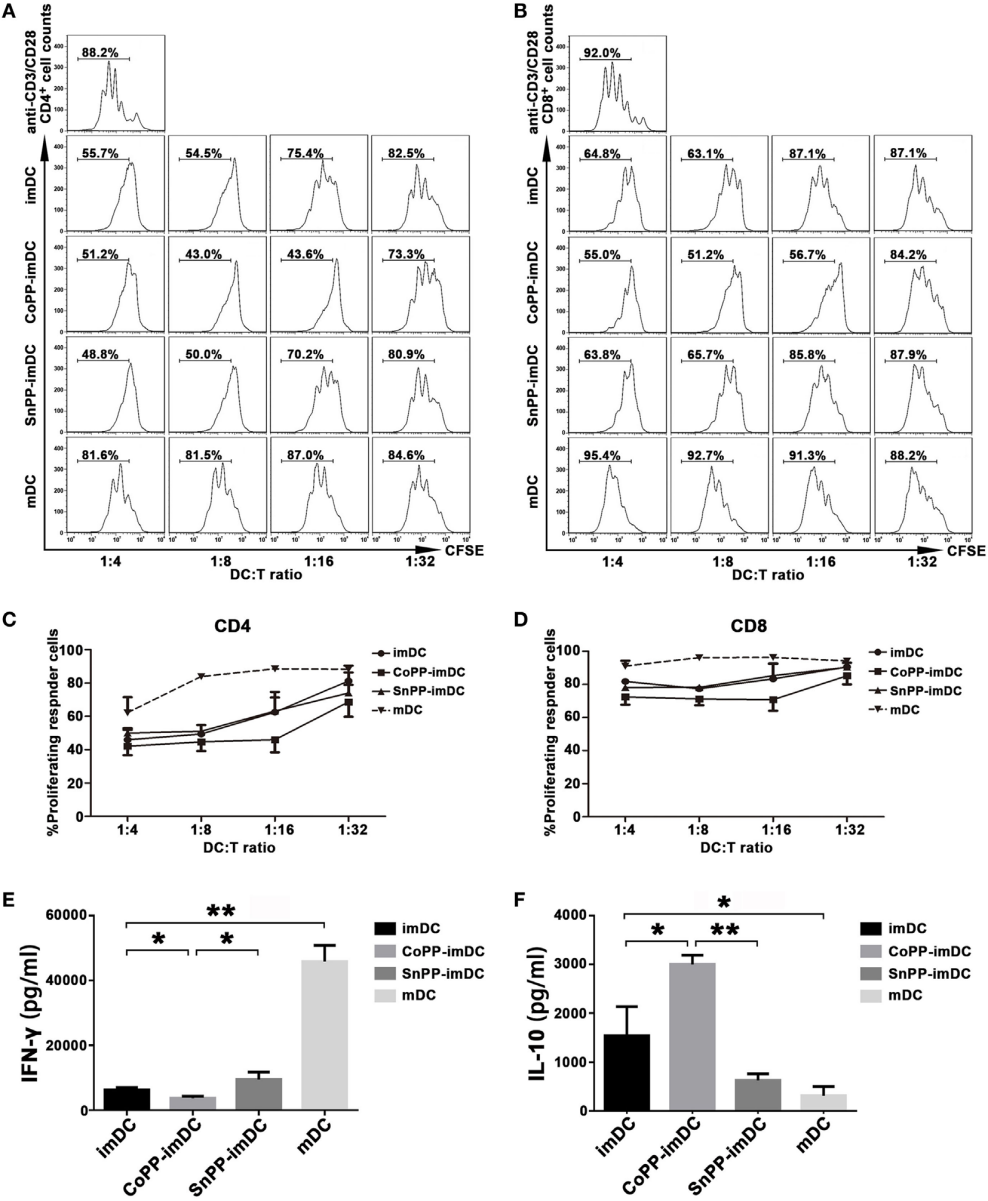
Upregulation of heme oxygenase-1 enhances the ability of immature dendritic cells (imDCs) to inhibit allogeneic T-cell proliferation stimulated by anti-CD3/CD28 Abs. CFSE-labeled lymphocytes from C57BL/6 mice were stimulated with anti-CD3ε and anti-CD28 Abs (2 µg/ml) to achieve profound cell proliferation. Graded doses of untreated imDCs, cobalt protoporphyrin-pretreated imDCs, SnPP-pretreated imDCs, or mDCs from BALB/c mice were added to the lymphocyte stimulation system at different ratios (1:4, 1:8, 1:16, and 1:32). Three days later, CD4^+^ and CD8^+^ T-cell proliferation was separately evaluated by FACS analysis. **(A,B)** Representative FACS results of three independent experiments for CD4^+^ **(A)** and CD8^+^ **(B)** T-cell proliferation. Numbers within the graph denote the percentage of proliferated cells. **(C,D)** The average percentages of proliferated CD4^+^ **(C)** and CD8^+^ **(D)** T cells. Data are shown as mean ± SD (*n* = 3 per group). **(E,F)** Supernatants were collected after 48 h from the lymphocyte stimulation system at a DC/T cell ratio of 1:16 and analyzed by ELISA to determine the levels of IFN-γ **(E)** and IL-10 **(F)**. Data are shown as means ± SD (*n* = 3 per group, *P < 0.05, **P < 0.01).

We next determined whether inducing high expression of HO-1 *in vitro* could improve the capacity of imDCs to actively suppress allogeneic T-cell proliferation. For this purpose, we stimulated CFSE-labeled lymphocytes from C57BL/6 mice with anti-CD3 and anti-CD28 Abs to achieve profound cell proliferation. Untreated imDCs, CoPP-pretreated imDCs, SnPP-pretreated imDCs, or mDCs from BALB/c mice were added to the lymphocyte stimulation system at different ratios (1:4, 1:8, 1:16, and 1:32). Three days later, proliferation of CD4^+^ and CD8^+^ T cells was evaluated by FACS analysis. As shown in Figures 3A–D, anti-CD3/CD28 Abs induced significant proliferation of both CD4^+^ and CD8^+^ T cells, and this proliferation was nearly unaffected by the cocultured allogeneic mDCs. In contrast, untreated imDCs and SnPP-pretreated imDCs were capable of inhibiting the proliferation of both CD4^+^ and CD8^+^ T cells in a similar manner, and CoPP-pretreatment of imDCs further improved the inhibitory effects on T-cell proliferation.

We also investigated cytokine production in supernatants from the lymphocyte stimulation system, adding different types of DCs at a ratio of 1:16. As compared to mDCs, coculture with untreated imDCs significantly reduced IFN-γ production (*P* < 0.01) and increased IL-10 production (*P* < 0.05) (Figures 3E,F). When the cocultured imDCs were pretreated with CoPP, a further reduction in IFN-γ production was observed; in contrast, IL-10 was further increased (*P* < 0.05, vs. the untreated imDC group), and SnPP had almost no effect (Figures 3E,F).

### Adoptive Transfer of CoPP-Pretreated imDCs Is More Efficient Than That of Untreated imDCs in Prolonging Heart Allograft Survival

**Figure 4:**
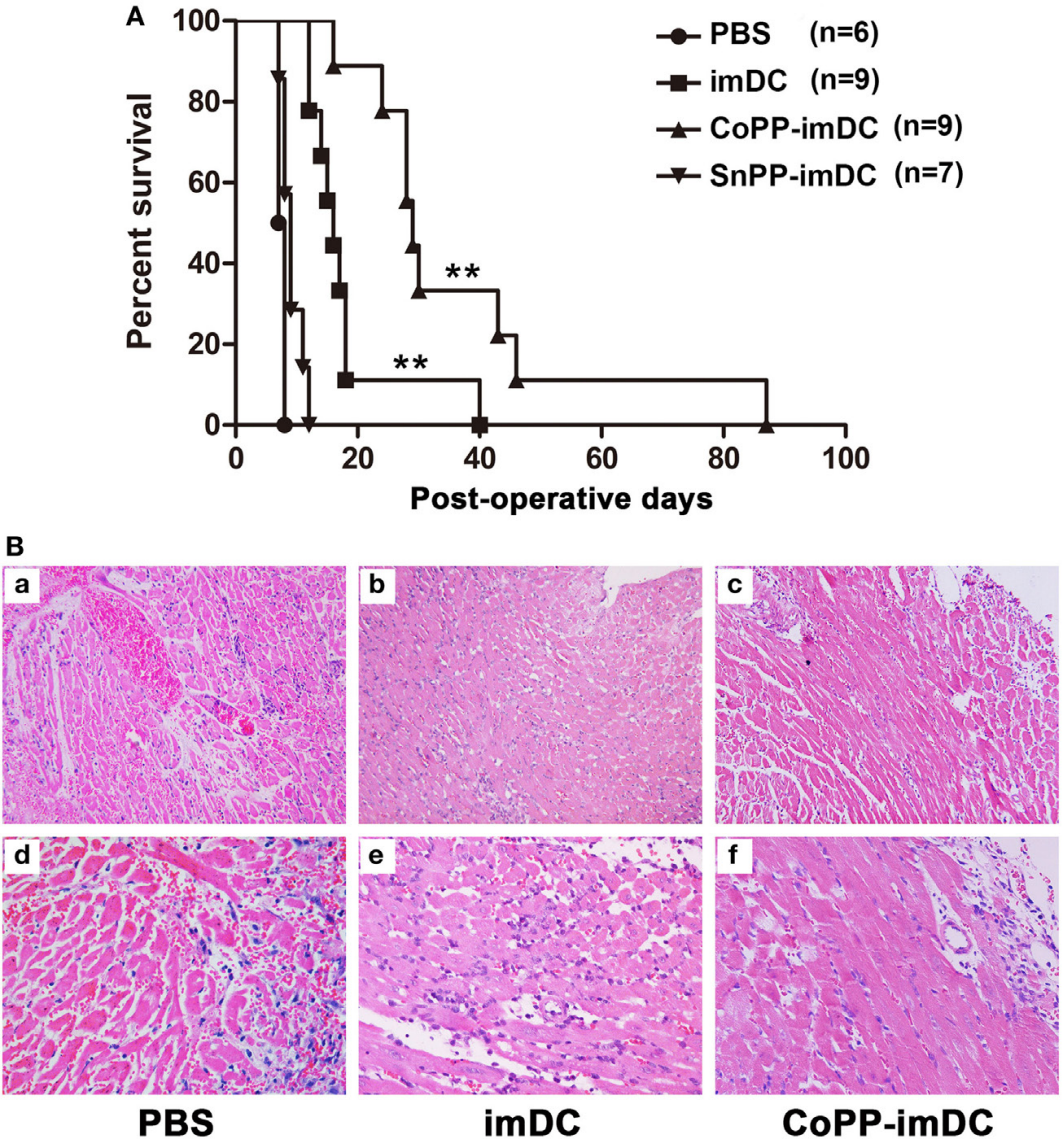
Donor-derived HO-1^hi^ immature dendritic cells (imDCs) are more efficient in modulating allograft rejection and prolonging heart allograft survival. Untreated, cobalt protoporphyrin (CoPP)-pretreated, or SnPP-pretreated BALB/c mouse-derived imDCs (5 × 10^6^) were injected intravenously into C57BL/6 recipients 7 days before cardiac transplantation. As a control, recipient mice received no cell transfusion, and only injection with the same volume of PBS. **(A)** The kinetics of cardiac allograft survival rates for all study groups are shown (**P < 0.01, untreated imDC group vs. both control group and SnPP-pretreated imDC group; CoPP-pretreated imDC group vs. untreated imDC group; *n* = 6–9 per group); **(B)** hematoxylin and eosin staining was performed to assess the pathological changes in allografts harvested at day 7 posttransplant in the PBS control group, untreated imDC group, and CoPP-pretreated imDC group (a–c: magnification, 100×; d–f: magnification, 200×).

To investigate whether donor-derived imDCs with high *in vitro*-induced HO-1 expression could be more efficient in modulating allograft rejection and prolonging heart allograft survival, we injected 5 × 10^6^ untreated, CoPP-pretreated, or SnPP-pretreated BALB/c mice-derived imDCs intravenously into C57BL/6 recipients of BALB/c cardiac allografts 7 days before transplantation. As a control, heart allograft recipients received no cell transfusion (only PBS injection). Adoptive transfer of donor-derived untreated imDCs significantly prolonged cardiac allograft survival when compared to the PBS control group (median = 16 days, *n* = 9; vs. PBS control group: median = 7.5 days, *n* = 6; *P* < 0.01) (Figure 4A). Interestingly, adoptive transfer of CoPP-pretreated imDCs further extended the prolongation of allograft survival (median = 29 days, *n* = 9; vs. untreated imDCs group: *P* < 0.01) (Figure 4A). In contrast, when the transfused imDCs were pretreated *in vitro* with SnPP to block HO-1 production, the prolongation of allograft survival resulting from imDC treatment was almost totally abrogated (median = 9 days, *n* = 7; vs. untreated imDCs group: *P* < 0.01) (Figure 4A). These data demonstrate that *in vitro* induction of high HO-1 expression can markedly enhance the capacity of donor-derived imDCs to prolong allograft survival *in vivo*.

Next, cardiac allografts (*n* = 3/group) were harvested at 7 days after transplantation, and H&E staining was performed to assess the pathological changes associated with acute allograft rejection. Allografts in the PBS control group developed signs of severe acute rejection at posttransplant day 7 (Figure 4B), characterized by diffuse predominantly mononuclear cell infiltrates, myocardial interstitial edema, and myocardial hemorrhage. In contrast, mice transfused with either untreated or CoPP-pretreated donor-derived imDCs demonstrated significant attenuation of these pathological changes in heart allografts at 7 days after transplantation, especially in the CoPP-pretreated group (Figure 4B).

### Donor-Derived imDCs Are Mainly Localized to Recipient Spleens After Adoptive Transfer

**Figure 5:**
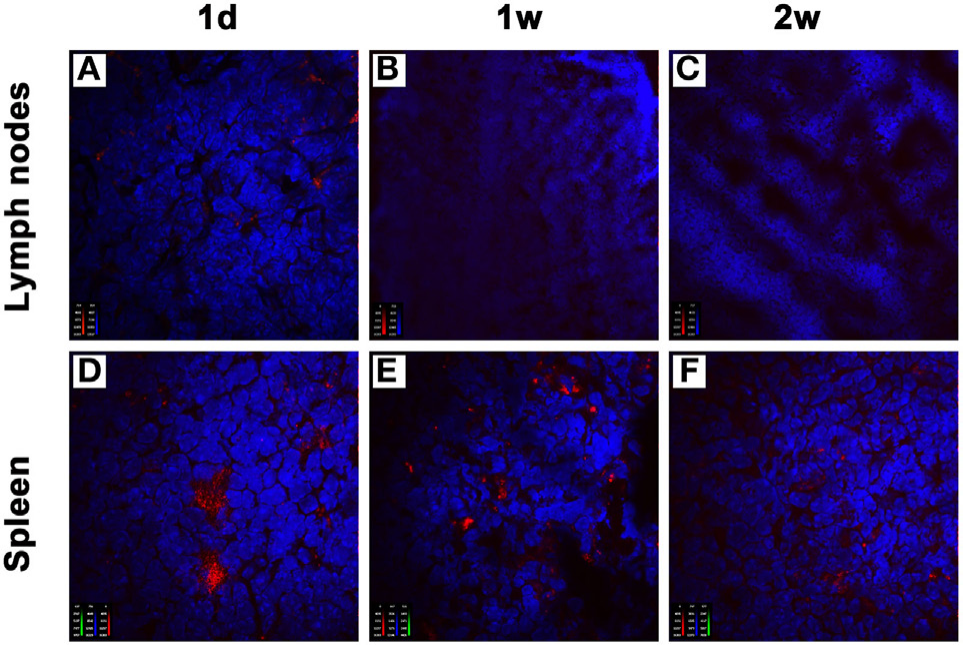
Donor-derived immature dendritic cells (imDCs) are mainly located in recipient spleens after adoptive transfer. Untreated BALB/c mouse-derived imDCs (5 × 10^6^) labeled with CellVue Claret (red) were intravenously injected into C57BL/6 recipients. At 1, 7, and 14 days after the injection, mesenteric lymph nodes **(A–C)** and spleens **(D–F)** were harvested to detect the localization of allogeneic imDCs. After staining with DAPI (blue), tissue sections were observed under a confocal imaging system. The photographs shown are representative of three independent experiments.

To understand the compartmentalization of the inhibitory effect of donor-derived imDCs in recipients, we analyzed their localization after an intravenous injection. BALB/c mice-derived imDCs labeled with CellVue Claret were injected into C57BL/6 mice *via* their tail veins. At 1, 7, or 14 days after adoptive transfer, the mesenteric lymph nodes and spleens were harvested from the C57BL/6 mice (*n* = 3/time point). After staining with DAPI, tissue sections were observed under a confocal imaging system. One day after cell transfusion, large numbers of donor-derived DCs were found in the spleen, and only small numbers in the mesenteric lymph nodes (Figures 5A,D). At 7 or 14 days after adoptive transfer, the presence of transfused DCs was still observed in the spleen, but not in the mesenteric lymph nodes (Figures 5B,C,E,F). These results suggest that the spleen may be the major site at which the adoptively transferred donor imDCs exert their immunoregulatory effects on recipient lymphocytes.

### *In Vitro* Upregulation of HO-1 Enables Donor-Derived imDCs to Survive Longer in Allogeneic Recipients After Adoptive Transfer

**Figure 6:**
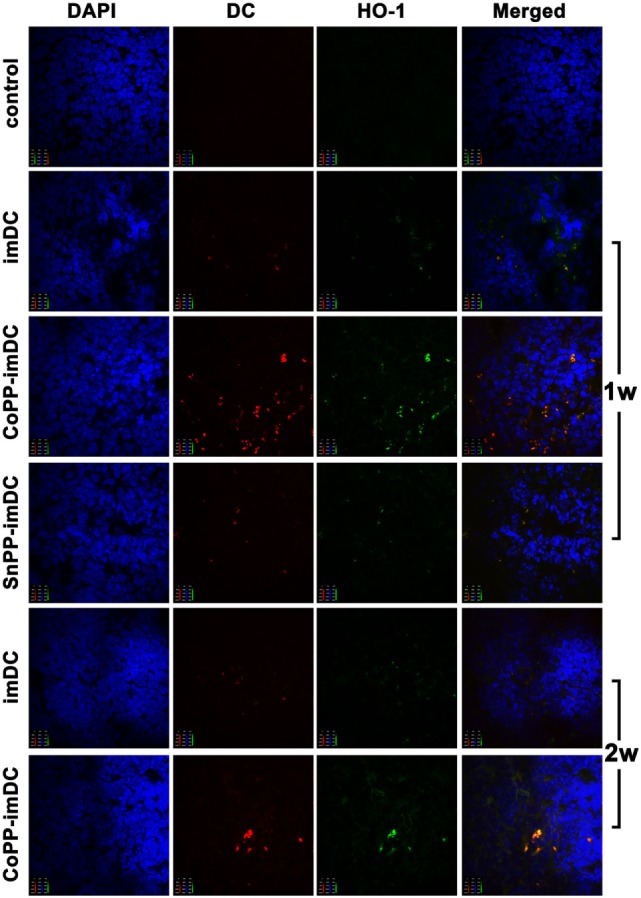
The survival of donor-derived dendritic cells in recipient spleen and their expression of heme oxygenase-1 (HO-1) after adoptive transfer. After being labeled with CellVue Claret, untreated, cobalt protoporphyrin-pretreated-, or SnPP-pretreated BALB/c mouse-derived immature dendritic cells (5 × 10^6^) were injected intravenously into C57BL/6 mice. Seven or 14 days later, the spleens were harvested for immunofluorescent detection of CellVue Claret (red), HO-1 expression (green), and DAPI (red). Representative photographs were captured under a confocal imaging system (magnification, 600×; *n* = 3 per group).

We next investigated whether *in vitro*-induced high HO-1 expression in donor-derived imDCs could prolong DC survival, and whether the high HO-1 expression status could be maintained in allogeneic recipients after adoptive transfer. After labeling the cells with CellVue Claret, we intravenously injected 5 × 10^6^ untreated, CoPP-pretreated, or SnPP-pretreated BALB/c mouse-derived imDCs into C57BL/6 mice. After 7 and 14 days, we harvested the spleens (*n* = 3/group) for immunofluorescent detection of CellVue Claret-labeled DCs (red), HO-1 expression (green), and DAPI (purple). Color images were captured using a confocal imaging system. At 7 days after adoptive transfer, a few scattered donor-derived DCs with HO-1 positive expression were found in the spleens of mice receiving transfusion with untreated or SnPP-pretreated imDCs, whereas large numbers of CellVue Claret-labeled DCs with high HO-1 expression were present in the spleens of mice who had received an injection of CoPP-pretreated imDCs (Figure 6). At 14 days after cell transfusion, a certain number of CoPP-pretreated imDCs with high HO-1 expression were still observed in the spleen, but only a few untreated imDCs could be seen (Figure 6). These results indicate that the induction of high HO-1 expression *in vitro* can prolong the survival of donor-derived imDCs in recipients after adoptive transfer; furthermore, these cells seemed to be able to retain their HO-1 overexpression during their prolonged survival.

### Donor-Derived High HO-1-Expressing imDCs Are More Efficient Than Untreated imDCs in Suppressing Both T Cell and B Cell Responses in Cardiac Allograft Recipients

**Figure 7:**
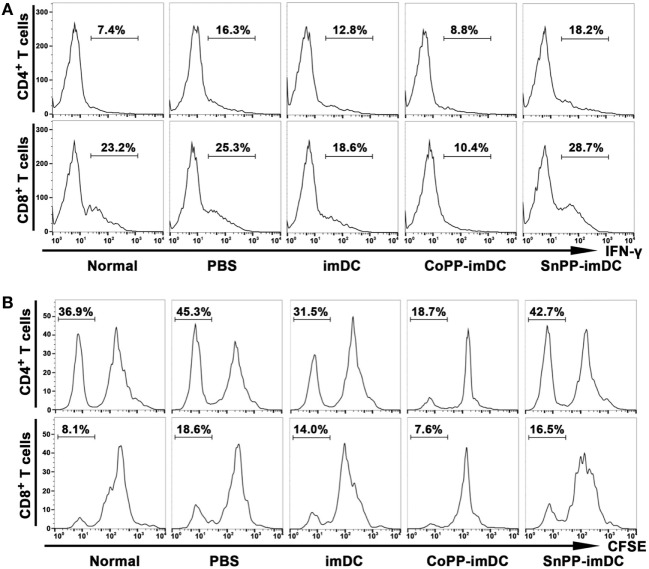
Adoptive transfer of donor-derived HO-1^hi^ immature dendritic cells (imDCs) is more efficient in suppressing the activation and proliferation of recipient lymphocytes. Untreated, cobalt protoporphyrin-pretreated, or SnPP-pretreated donor-derived imDCs (5 × 10^6^) were injected intravenously into recipient mice, and heart transplantation was performed 7 days later. As a control, recipient mice received no cell transfusion, and only an injection of the same volume of PBS. The splenocytes were isolated from normal C57BL/6 mouse spleens or recipient spleens collected 7 days after heart transplantation (*n* = 3 per group). **(A)** The expression of IFN-γ in CD4^+^ or CD8^+^ T cells was detected by flow cytometry. Representative FACS results of three independent experiments are shown. **(B)** mixed leukocyte reaction (MLR) was performed using irradiated donor splenocytes as stimulator cells and CFSE-labeled recipient splenocytes as responder cells. The proliferation of recipient CD4^+^ and CD8^+^ T cells was measured by FACS after a 7-day MLR. Representative FACS results of three independent experiments are shown.

To further investigate whether adoptively transferred HO-1 high-expressing imDCs are more efficient than controls in inhibiting allogenic lymphocyte activation and proliferation in heart allograft recipients, we intravenously injected donor-derived imDCs without any pretreatment or pretreated with CoPP or SnPP into the recipient mice and performed heart transplantation 7 days later. The recipient spleens were collected 7 days after heart transplantation, and IFN-γ expression in T cells of splenocytes was measured by FACS. As compared to the PBS control group without DC transfusion, adoptive transfer with untreated donor-derived imDCs significantly reduced the IFN-γ expression in both CD4^+^ and CD8^+^ T cells from recipient spleens (Figure 7A). When adoptively transferred imDCs were pretreated with CoPP, a further reduction in IFN-γ expression in recipient T cells was observed; SnPP, on the other hand, had almost no inhibitory effect (Figure 7A).

We also collected recipient spleens and isolated splenocytes to perform MLR on day 7 after heart allotransplantation. Irradiated donor (BALB/c) splenocytes were used as stimulator cells, and CFSE-labeled recipient (C57BL/6) splenocytes as responder cells. The proliferation of recipient CD4^+^ and CD8^+^ T cells was measured by FACS after a 7-day MLR. As compared to the PBS control group, adoptive transfer with untreated donor-derived imDCs significantly attenuated the *in vitro* proliferation of both CD4^+^ and CD8^+^ T cells from recipient spleens, whereas adoptive transfer with CoPP-pretreated imDCs further decreased the proliferation of recipient T cells (Figure 7B). In contrast, transfusion of SnPP-pretreated donor-derived imDCs had almost no inhibitory effect on recipient T-cell proliferation after the *in vitro* alloantigen stimulation (Figure 7B).

**Figure 8:**
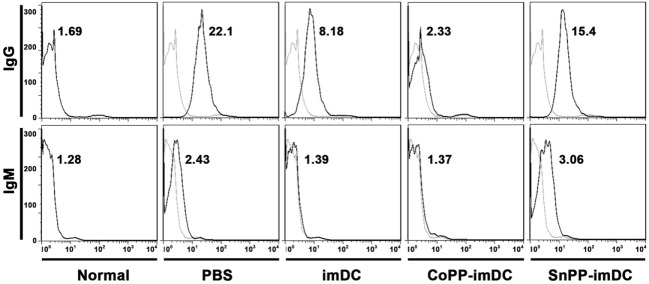
Donor-derived HO-1^hi^ immature dendritic cells (imDCs) are more potent in attenuating anti-donor humoral responses in cardiac allograft recipients. Untreated, cobalt protoporphyrin-pretreated, or SnPP-pretreated BALB/c mouse-derived imDCs were adoptively transferred to C57BL/6 recipients 7 days before cardiac transplantation. As a control, recipient mice received no cell transfusion, and only injection with the same volume of PBS. The sera were collected from normal C57BL/6 mice or recipient mice on day 7 after heart transplantation (*n* = 3 per group). The circulating anti-donor IgG and IgM levels were detected by flow cytometry using BALB/c splenocytes as target cells. Data expressed are the Gmean values. Representative FACS results of three independent experiments are shown.

To access the humoral responses against donor antigens after DC transfusion, we collected recipient sera on day 7 after heart allotransplantation. After incubation of the sera with splenocytes of BALB/c origin at same dilution, the binding of IgG and IgM on donor cells was analyzed by flow cytometry. As compared to normal controls, recipient mice in PBS control group generated high levels of anti-donor IgG and slightly increased anti-donor IgM (Figure 8). Adoptive transfer with untreated donor-derived imDCs significantly attenuated the generation of alloantibodies, whereas adoptive transfer with CoPP-pretreated imDCs further decreased the alloantibody production (Figure 8). In contrast, transfusion of SnPP-pretreated donor-derived imDCs had almost no inhibitory effect on the recipient B-cell responses (Figure 8).

The results described above suggest that adoptively transferred donor-derived high HO-1-expressing imDCs show increased potency in inhibiting both T-cell and B-cell responses in cardiac allograft recipients.

## Discussion

An increasing number of experimental studies have shown that immunotherapy with donor-derived imDCs, expanded *in vitro* and rendered immunosuppressive by pharmacologic or genetic methods, are capable of promoting transplant tolerance in rodent models (13). Before tolerogenic DC-based therapy is used clinically, it is essential to identify how imDCs can be prevented from acquiring pro-inflammatory abilities, in order to offer a safe therapeutic option in humans. Moreover, in order to improve the therapeutic results, it is important to obtain more powerful and persistently tolerogenic DCs. In the present study, by using a stringent mouse cardiac allotransplant model, we have demonstrated, for the first time, that *in vitro* induction of high HO-1 expression enables donor-derived imDCs to survive longer and exert more potent immunoregulatory effects in allogenic recipients after adoptive transfer, resulting in a significant prolongation of allograft survival.

Heme oxygenase-1 plays important roles in regulating DC functions, such as DC maturation, differentiation, homing, and mediating T-cell responses (17, 18, 21). Whether all DC subsets can express HO-1 or not is an important question. Chauveau and coworkers have reported that human monocyte-derived imDCs and several, but not all, freshly isolated rat splenic DC subsets and rat bone marrow-derived imDCs spontaneously express HO-1 and that HO-1 expression markedly decreases during DC maturation induced *in vitro* (18). In the present study, we report similar findings concerning *in vitro*-generated mouse DC subsets, showing that imDCs (adherent BMDCs) express HO-1, whereas mDCs obtained through the treatment of non-adherent BMDCs with LPS only exhibit very weak expression of HO-1. Taken together, these data indicate that HO-1 is mainly spontaneously expressed in imDCs, but not in mDCs, suggesting that HO-1 may be closely associated with the function of imDCs. In addition, since HO-1 is capable of being induced by several specific chemical inducers (such as CoPP), we also investigated whether the DC maturation status has any influence on the HO-1 upregulation induced by CoPP. We have now demonstrated that CoPP treatment can only induce significantly enhanced expression of HO-1 in mouse imDCs, but not in mDCs. Why fully mature DCs almost entirely lack the capacity to express HO-1 needs to be further investigated.

An important consideration regarding tolerogenic DC-based therapy is that of ensuring that these imDCs are able to resist maturation in response to pro-inflammatory stimuli. imDCs incubated with agents such as vitamin D3 (VitD3), 1 α,25-dihydroxyvitamin D3, IL-10, and rapamycin have been reported to gain this capacity (11, 22–25). Given that HO-1 expression almost disappears after imDCs become fully mature, induction of high HO-1 expression may prevent imDCs from maturating under pro-inflammatory stimuli. Chauveau and coworkers have demonstrated that induction of HO-1 expression with CoPP in human and rat imDCs inhibits LPS-induced maturation (18). In present study, we also demonstrated that the induction of high HO-1 expression in mouse imDCs results in a significant blockade of LPS-induced phenotypic maturation of DCs. Furthermore, we have demonstrated, for the first time, that *in vitro*-induced HO-1^hi^ imDCs have increased potency as immunoregulatory DCs, as evidenced by their enhanced ability to inhibit allogeneic T-cell proliferation stimulated by anti-CD3/CD28 Abs. These results suggest that *in vitro*-generated HO-1^hi^ imDCs may be excellent candidates for use in establishing transplant tolerance.

To address the question of whether *in vitro*-generated HO-1^hi^ imDCs are more efficient in modulating alloreactive immune responses and thereby prolong allograft survival, we performed an adoptive transfer study using a stringent mouse cardiac allotransplant model (BALB/c-to-C57BL/6). Since compromised graft survival prolongation was observed when donor-derived DCs were transfused earlier or later than 7 days before allotransplantation (8), DCs were usually intravenously injected into the recipients 1 week before transplantation in the majority of studies that explore the tolerogenic effects of donor-derived DCs (7). Therefore, we chose the same timepoint to perform DC adoptive transfer according to published procedures (8, 26). The extent of the graft prolongation that we observed with HO-1^hi^ imDCs [mean survival time (MST), 29 days] was superior to that obtained with conventional imDCs (MST, 19 days). Interestingly, when the transfused imDCs were pretreated *in vitro* with SnPP to block HO-1 activity, the prolongation of allograft survival resulting from imDCs therapy was almost totally abrogated (MST, 9 days). In addition, we found that the adoptively transferred donor-derived HO-1^hi^ imDCs were more efficient than conventional imDCs in suppressing the activation and proliferation of recipient T cells in response to alloantigen, as well as the production of alloantibodies by recipient B cells. These results indicate that HO-1 plays a critical role in the *in vivo* immunoregulatory effects of donor-derived imDCs after adoptive transfer in the context of transplantation. To our knowledge, this is the first *in vivo* study to evaluate the effectiveness of therapy with HO-1^hi^ imDCs.

To understand the mechanisms by which donor-derived imDCs play a role in recipients, we analyzed their localization after intravenous injection and investigated whether high HO-1 expression induced *in vitro* in donor-derived imDCs could prolong DC survival and whether the status of high HO-1 expression could be maintained in allogeneic recipients after the adoptive transfer. We found that donor-derived imDCs were mainly located in the recipient spleens instead of the mesenteric lymph nodes at 1, 7, or 14 days after adoptive transfer, a finding that is in agreement with previous reports showing that DCs injected intravenously preferentially migrate to the spleen (9, 27, 28). These results suggest that the spleen may be the major place where the adoptively transferred donor imDCs exert their immunoregulatory effects on recipient lymphocytes. More importantly, we found that *in vitro*-induced high HO-1 expression can prolong the survival time (up to 2 weeks) of donor-derived imDCs in recipient mice after adoptive transfer, and these cells seemed to be able to maintain their HO-1 overexpression status during this time. Since the adoptively transferred recipient DCs were expected to survive for longer period of time (up to 2 weeks) than the injected donor DCs (6, 9), the donor-derived HO-1^hi^ imDCs in our study were capable of surviving in recipient mice as long as the adoptively transfused recipient DCs. Divito et al. have reported that intravenously administered, maturation-resistant allogeneic DCs function as antigen-transporting cells rather than antigen-presenting cells to prolong allograft survival (29). Whether our allogeneic HO-1^hi^ imDCs exert their immunoregulatory effects *via* direct or indirect pathways needs to be further investigated. Additionally, it has been reported that recipient-derived imDCs are superior than donor-derived imDCs in prolonging graft survival in a rat cardiac allotransplant model (9), and that the *in vivo* tolerogenic effect of recipient-derived imDCs is at least in part mediated by HO-1 (17). Whether recipient-derived HO-1^hi^ DCs are more potent than donor-derived HO-1^hi^ DCs in prolonging allograft survival in our murine cardiac transplant model is also worthy of further study.

The cellular mechanisms of how HO-1 induces DC immunoregulatory function are not very clear. Recent evidences have shown that (1) both HO-1 and CO can decrease endosome-lysosome fusion and inhibit soluble antigen presentation by DCs to T cells (30); (2) CO is able to regulate DC immunogenicity by a mitochondria-dependent mechanism (31). Therefore, HO-1 may prevent T cell responses by producing carbon monoxide (CO) and impairing DC immunogenicity. Additionally, other possible mechanisms by which induction of HO-1 overexpreesion promotes DC immunoregulatory function includes: (1) upregulation of PD-L1, a known inhibitory receptor, which may reduce antigen-specific T cell proliferation (32); (2) the retainment of IL-10 secretion in DCs, which may promote formation of type 1 regulatory cells (18, 33); (3) the ability to express TGF-β, accompanied by low levels of IL-6, may favor Treg differentiation over Th17 (32, 34).

In conclusion, we have identified *in vitro*-induced HO-1^hi^ imDCs as a type of more potent and mature-resistant immunoregulatory DC. We have also demonstrated, for the first time, that adoptively transferred donor-derived HO-1^hi^ imDCs can survive longer and sustain their HO-1 high expression status in allogeneic recipients, resulting in a significant prolongation of allograft survival in a stringent mouse cardiac allotransplant model. HO-1^hi^ imDC-based cell therapy may represent an antigen-specific and cost-effective novel strategy to induce transplant tolerance.

## Ethics Statement

Animals were maintained according to the Guide for the Care and Use of Laboratory Animals of the National Institutes of Health, and the protocol was approved by the Ethical Committee on Animal Experiments of Tongji Medical College, Huazhong University of Science and Technology.

## Author Contributions

YZ, YJ, and GC designed the study and interpreted the data; wrote the manuscript; and analyzed the data. YZ, YJ, LW, SC, XH, BX, and GZ performed the experiments; JY and GC supervised the experiments. All authors revised the work and gave final approval of the version to be published.

## Conflict of Interest Statement

The authors declare that the research was conducted in the absence of any commercial or financial relationships that could be construed as a potential conflict of interest.

## Abbreviations

HO-1, heme oxygenase-1; imDCs, immature dendritic cells; BMDCs, bone marrow-derived DCs; CoPP, cobalt protoporphyrin; mDCs, mature dendritic cells.
