# Supplementary material for: Upregulation of Heme Oxygenase-1 Endues Immature Dendritic Cells With More Potent and Durable Immunoregulatory Properties and Promotes Engraftment in a Stringent Mouse Cardiac Allotransplant Model
Source: Front Immunol. 2018 Jul 2;9:1515. doi: 10.3389/fimmu.2018.01515 (PMC6036127; doi:10.3389/fimmu.2018.01515)
Supplement: Supplementary file 1 [file Data_Sheet_1.docx]

Supplementary Material

Upregulation of heme oxygenase-1 endues immature dendritic cells with more potent and durable immunoregulatory properties and promotes engraftment in a stringent mouse cardiac allotransplant model

Yue Zhao^1, 5,6, †^, Yu Jia^1,2,†^, Lu Wang^1,3,4^, Song Chen^1,3,4^, Xia Huang^1,3,4^, Bingyang Xu^1^, Guangyuan Zhao^1^, Ying Xiang^1,3,4^, Jun Yang^1,3,4^, Gang Chen^1,3,4,*^

*** Correspondence:** Dr. Gang Chen: [gchen@tjh.tjmu.edu.cn](mailto:gchen@tjh.tjmu.edu.cn)

## Supplementary Figure

**
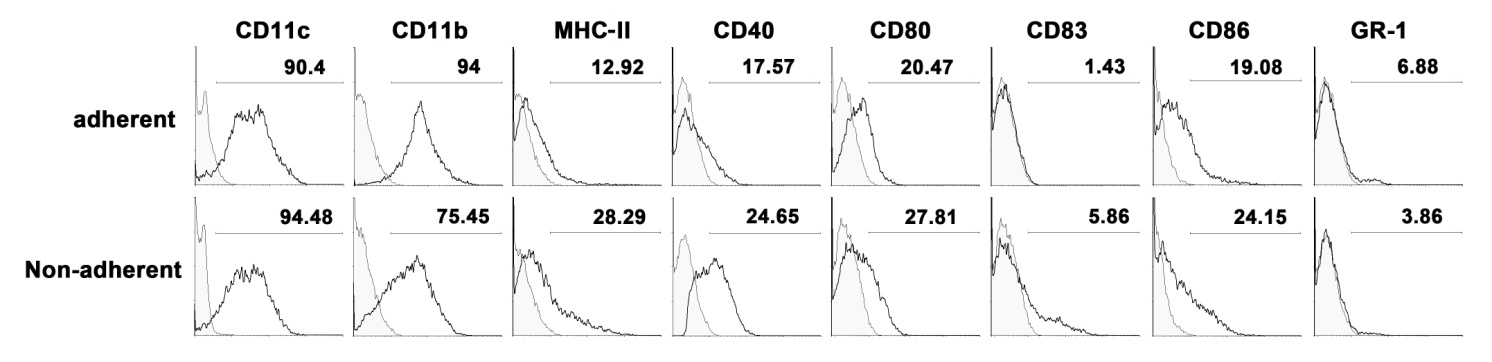
**

**Supplemental Figure 1. Phenotype of murine BM-derived DCs**

Adherent or non-adherent DCs derived from murine BM cells were characterized by cytofluorometry on day 10 of culture. Black lines in histograms show the expression of different markers in adherent and non-adherent BMDCs gated on live cells. Markers used were CD11c, CD11b, MHC-II, CD40, CD80, CD83, CD86, and GR1. Gray lines represent isotype controls. Numbers in quadrants indicate the percentage of positive cells. Data are representative of three independent experiments.
